# Bibliometric and visualized analysis of ultrasound combined with microbubble therapy technology from 2009 to 2023

**DOI:** 10.3389/fphar.2024.1418142

**Published:** 2024-07-25

**Authors:** Bin Tu, Yan Li, Wen Wen, Jian Liu

**Affiliations:** ^1^ Department of Ultrasound, First Affiliated Hospital of Chengdu Medical College, Chengdu, China; ^2^ Department of Nephrology, The Affiliated Hospital of Southwest Medical University, Luzhou, Sichuan, China

**Keywords:** ultrasound, microbubble, bibliometric analysis, collaborative network, hot spots

## Abstract

**Background:**

In recent years, with the rapid advancement of fundamental ultrasonography research, the application of ultrasound in disease treatment has progressively increased. An increasing body of research indicates that microbubbles serve not only as contrast agents but also in conjunction with ultrasound, enhancing cavitation effects and facilitating targeted drug delivery, thereby augmenting therapeutic efficacy. The objective of this study is to explore the current status and prevailing research trends in this field from 2009 to 2023 through bibliometric analysis and to forecast future developmental trajectories.

**Methods:**

We selected the Science Citation Index Expanded (SCI-Expanded) from the Web of Science Core Collection (WOSCC) as our primary data source. On 19 January 2024, we conducted a comprehensive search encompassing all articles and reviews published between 2009 and 2023 and utilized the bibliometric online analysis platform, CiteSpace and VOSviewer software to analyze countries/regions, institutions, authors, keywords, and references, used Microsoft Excel 2021 to visualize the trends of the number of articles published by year.

**Results:**

Between 1 January 2009, and 31 December 2023, 3,326 publications on ultrasound combined with microbubble therapy technology were included. There were a total of 2,846 articles (85.6%) and 480 reviews (14.4%) from 13,062 scholars in 68 countries/regions published in 782 journals. China and the United States emerged as the primary contributors in this domain. In terms of publication output and global institutional collaboration, the University of Toronto in Canada has made the most significant contribution to this field. Professor Kullervo Hynynen has achieved remarkable accomplishments in this area. *Ultrasound in Medicine and Biology* is at the core of the publishing of research on ultrasound combined with microbubble therapy technology. Keywords such as “sonodynamic therapy,” “oxygen,” “loaded microbubbles” and “Alzheimer’s disease” indicate emerging trends in the field and hold the potential to evolve into significant areas of future investigation.

**Conclusion:**

This study provides a summary of the key contributions of ultrasound combined with microbubble therapy to the field’s development over the past 15 years and delves into the historical underpinnings and contemporary trends of ultrasound combined with microbubble therapy technology, providing valuable guidance for researchers.

## 1 Introduction

Ultrasound, as a widely utilized, economically feasible, safe, effective, and real-time imaging modality in clinical diagnosis, spans almost all medical disciplines. In recent years, with the rapid advancement of ultrasonographic foundational research, in addition to its role in disease imaging diagnostics, its application in disease treatment has been progressively explored. Therapeutic ultrasound enables the delivery of energy into deep tissue layers to induce various biological effects, thereby altering physiological structures or functions (Zhang et al., 2023).

It is well known that ultrasound can directly induce thermal, mechanical, and cavitation effects. Among these effects, the cavitation effect is one of the main biological effects of ultrasound therapy and describes the impact of ultrasound on the formation, growth, oscillation, and collapse of microbubbles in a medium, including stable cavitation and inertial cavitation (Ahmadi et al., 2012). The intensity of the ultrasound cavitation effect is related to several parameters, such as the mechanical index, duty cycle, frequency, acoustic pressure, pulse length, and pulse repetition frequency (Lin et al., 2017). Stable cavitation refers to the oscillation of microbubbles exposed to low mechanical index ultrasound, causing the microbubbles to expand or compress, thereby exerting thrust or tension on the surrounding environment and resulting in microflows. An oscillation disturbance to the surrounding liquid or direct action on tissues causes physiological changes in the interaction (Ahmadi et al., 2012). Inertial cavitation refers to the continuous oscillation and enlargement of microbubbles at a high mechanical index, which eventually rupture after reaching a certain size (approximately twice the initial radius), generating shockwaves and microjets, applying high pressure to the surrounding medium, and possibly leading to transient temperature elevation and the formation of free radicals (Wells, 1977; Ashokkumar and Grieser, 2004; Yang and Church, 2005). Upon irradiation with ultrasound of a certain energy, the pulse emitted by ultrasound itself can increase the permeability of the cell membrane, and the combined application of ultrasound with microbubbles can enhance the cavitation effect.

Microbubbles, which are typically composed of a thin stabilizing coating, such as lipids, albumin, or polymers, encase an inflatable core, such as air or perfluorocarbons. With an average size ranging between 1–8 μm, these microbubbles can traverse the pulmonary capillary bed (Goldberg et al., 1994; Polat et al., 2011; Kooiman et al., 2014). The cavitation effect of acoustically transferred microbubbles was elucidated and subsequently harnessed in the medical domain during the 20th century, evolving from mere imaging contrast agents to delivery carriers. Within these microbubbles, pharmaceutical payloads may be encapsulated, or specific antibodies or receptors may be tethered to their surface, facilitating targeted delivery to pathological sites such as tumors via antigen-antibody or ligand‒receptor interactions. This multifaceted approach not only augments local microbubble concentrations and enhances cavitation efficiency but also enables precise drug release through transient cavitation, thereby augmenting therapeutic efficacy against neoplastic or other maladies (Zhang et al., 2014). Thus, ultrasound combined with microbubble technology engenders novel therapeutic avenues, particularly in cancer treatment (Li et al., 2012; Boissenot et al., 2016; Sharma et al., 2023), opening of the blood‒brain barrier (BBB) (Poon et al., 2017; Li et al., 2023; Lim Kee Chang et al., 2023), gene/drug delivery (Boissenot et al., 2016; Kancheva et al., 2023; Shi et al., 2023), and the treatment of other diseases (Zhang et al., 2021; Liu et al., 2023; Zhu et al., 2023).

In scientific research, the number of published papers is a significant metric for assessing research contributions. Bibliometric analysis serves as a quantitative method employed to delineate the knowledge structure and developmental trends within specific domains while also evaluating research outcomes, productivity, and impact (Donthu et al., 2021). At present, bibliometrics has emerged as a crucial method for researchers to effectively acquire the latest advancements in a given research field, forecast research focal points, and evaluate developmental trajectories within that domain. In recent years, there have been several bibliometric analyses pertaining to ultrasound (Wu et al., 2021; Sridharan et al., 2023), yet there remains a dearth of research on quantity and quality concerning the field of ultrasound combined with microbubble therapy technology. Therefore, the objective of this study is to explore the current status and prevailing research focal points in this domain from 2009 to 2023 through bibliometric analysis and to forecast future developmental trends.

## 2 Methods

### 2.1 Data source and retrieval strategies

**FIGURE 1:**
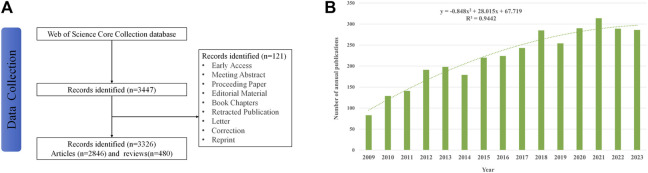
**(A)** Flowchart of literature selection; **(B)** trends in the growth of publications worldwide from 2009 to 2023. Each bar shows the number of publications per year. The dotted line represents the curve fitting for this trend.

Scopus and the WOS are the primary databases used for bibliometric research (Sood et al., 2022). In this study, we opted for the SCI-Expanded within the WOSCC as the primary data source. We conducted a comprehensive search of relevant publications from 2009 to 2023 on 19 January 2024. The retrieval strategy for this study was as follows: TS= (ultrasound OR ultrasonography OR ultrasonic OR sonoporation) AND TS= (microbubble*) AND TS= (therapy OR therapies OR treatment). The data categories are restricted to “articles” and “reviews.” A total of 3,326 publications meeting these criteria were included in the analysis, comprising 2,846 articles and 480 reviews, as depicted in Figure 1A.

### 2.2 Data analysis

We utilized various platforms for the analysis and visualization of WOSCC by extracting information from the collected databases, such as the bibliometric online analysis platform (https://bibliometric.com/), Microsoft Excel 2021, CiteSpace and VOSviewer software (Sood and Rawat, 2021).

Three different visualization maps with different meanings (network, density, and overlay visualization) and the total link strength (TLS) were generated by VOSviewer (version 1.6.20). TLS indicates the impact of a node on other nodes involved in the studies. VOSviewer was used to visualize country/regional cooperation, author co-authorship, and keyword co-occurrence. In the visual map, different nodes represent authors, journals, keywords, etc.; the node size indicates the number or frequency; the thickness of the line represents the strength of the link; and the node colors represent different clusters or times.

CiteSpace (6.2.R3) was used to analyze the included literature, including co-citation analysis of countries/regions and institutions, dual-map overlay of citations, timeline view, co-cited reference analysis, and references with the strongest citation bursts.

Microsoft Excel 2021 was used for visualize the trends of the number of articles published by year and draw the polynomial fitted curves of annual publication.

Moreover, the bibliometric online analysis platform was used to construct international cooperation networks between countries.

## 3 Results

### 3.1 Annual publication analysis

Between 1 January 2009, and 31 December 2023, a total of 3,326 publications on ultrasound combined with microbubble therapy technology were included. There were a total of 2,846 articles (85.6%) and 480 reviews (14.4%) from 13,062 scholars in 68 countries/regions published in 782 journals. As shown in Figure 1B, the number of publications on ultrasound combined with microbubble therapy has shown an overall increasing trend since 2009, with a significant correlation coefficient (R^2^ = 0.9442) between the two variables. The publication volume reached its peak in 2021 (314, 9.4%). Based on the current research situation, it is speculated that the field is in a stable development stage.

### 3.2 Global contributions and collaborations

From 2009 to 2023, the documents indexed by the Web of Science were published in 68 countries/regions. The 10 most productive countries/regions are listed in Table 1. Significantly, research on ultrasound combined with microbubble therapy technology is thriving in China and the USA. China (1,410 publications and 32,738 citations) was the most productive country, followed by the USA (963 publications and 34,677 citations) and Canada (265 publications and 10,566 citations). Although the total number of publications in Canada ranked third, its average number of citation value (value = 39.87) was greater than that of China (value = 23.22) and the USA (value = 36.01) and was second only to that of the Netherlands (value = 41.40). TLS indicates the impact of countries/regions’ published papers on other countries/regions involved in the studies. The USA had the greatest TLS (469 TLS), followed by China (321 TLS), and England (157 TLS).

**TABLE 1:** The 10 most productive countries/regions.

Rank	Country	Documents(n)	Citations	Average citations	TLS
1	China	1,410	32,738	23.22	321
2	USA	963	34,677	36.01	469
3	Canada	265	10,566	39.87	133
4	England	179	6,052	33.81	157
5	Japan	176	3,596	20.43	36
6	Germany	161	5,962	37.03	125
7	France	142	4,597	32.37	129
8	Netherlands	114	4,720	41.40	104
9	South Korea	99	2,117	21.38	38
10	Italy	90	2,377	26.41	75

TLS: total link strength.

**FIGURE 2:**
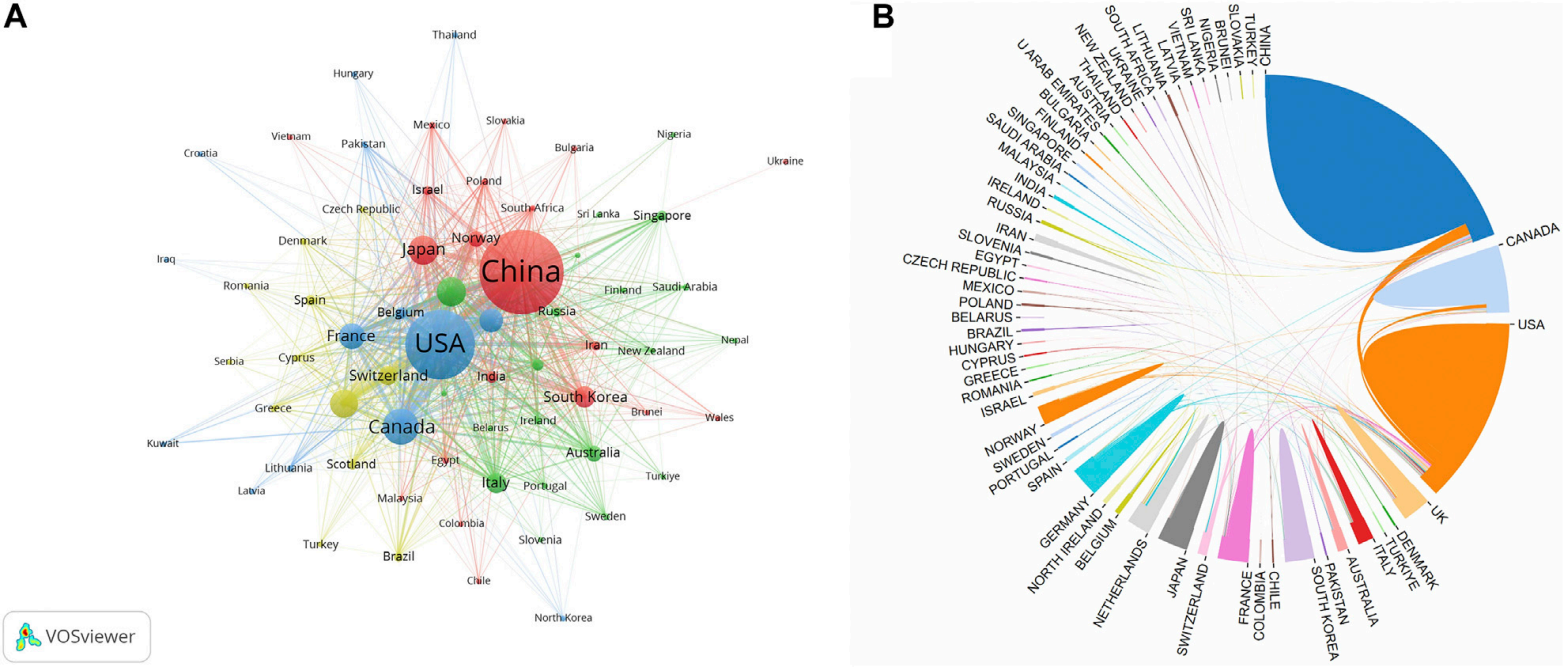
**(A)** The co-authorship network of countries/regions; **(B)** The international cooperation networks between countries.

Figure 2 shows the international cooperation among countries/regions. VOSviewer revealed global cooperation, with 66 countries/regions included in our analysis (Figure 2A). Each node in the network represented a country or region, with the node size corresponding to the number of publications. The lines connecting the nodes represent cooperation between countries. Figure 2B, generated by the bibliometric online analysis platform, shows the distribution and international collaboration of countries/regions engaged in research on ultrasound combined with microbubble therapy technology. The thickness of the line reflects the frequency of the cooperation. The thicker the line, the stronger the cooperation. The countries with the closest cooperation with other countries/regions were China and the USA.

### 3.3 Institutional contributions and collaborations

The present study involved a total of 2,274 institutions, with the top 10 contributing 29.95% (996 publications) of the total papers. The 10 most productive institutions are listed in Table 2, with half of them located in China and the remaining institutions located in Canada, the USA and England. The institution with the highest number of publications was the University of Toronto (181 papers and 8,492 citations), followed by Chongqing Medical University (160 papers and 5,409 citations) and the Chinese Academy of Sciences (123 papers and 5,772 citations). Among these top ten institutions. In addition, the top 3 institutions with the highest average citation value are Sunnybrook Research Institute (value = 47.53), the Chinese Academy of Sciences (value = 46.93) and the University of Toronto (value = 46.92).

**TABLE 2:** The 10 most productive institutions.

Rank	Institution	Country	Documents(n)	Citations	Average citations	TLS
1	Univ Toronto	Canada	181	8,492	46.92	344
2	Chongqing Med Univ	China	160	5,409	33.81	176
3	Chinese Acad Sci	China	123	5,772	46.93	262
4	Shanghai Jiao Tong Univ	China	105	2,359	22.47	136
5	Sunnybrook Res Inst	Canada	103	4,896	47.53	223
6	Stanford Univ	USA	72	3,294	45.75	86
7	Third Mil Med Univ	China	64	1,238	19.34	107
8	Univ Oxford	England	64	2,871	44.86	58
9	Sunnybrook Hlth Sci Ctr	Canada	63	2,593	41.16	144
10	Sun Yat Sen Univ	China	61	1,518	24.89	76

TLS: total link strength.

**FIGURE 3:**
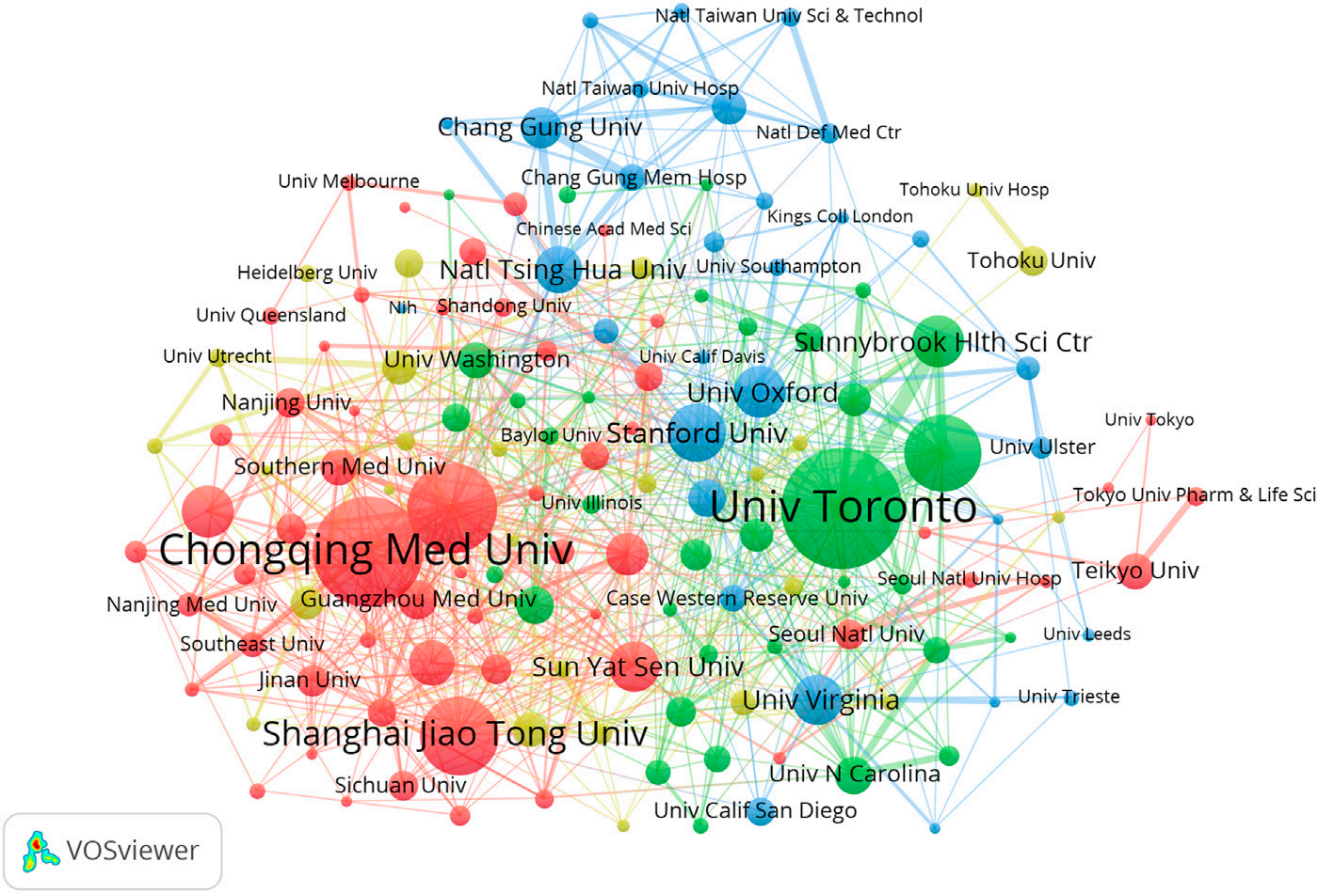
Co-operation network between institutions.

As shown in Figure 3, VOSviewer software was used to construct a collaboration network visualization map of the institutions with a minimal productivity of 10 publications (n = 148). Each node represents an institute. The scale of the node represents the number of publications. The lines between the nodes represent cooperation between the institutions. A thicker line between nodes represents a stronger cooperative relationship.

### 3.4 Leading authors and their contributions

A total of 13,062 researchers and 53,280 co-cited authors made contributions to the field of ultrasound combined with microbubble therapy technology between 2009 and 2023. The top 10 most productive authors are listed in Table 3. There are 6 authors from China, and the remaining 4 are from Canada, the United States, Germany, and England. Wang Zhigang (75 papers and 3,352 citations) from Chongqing Medical University in China is the most productive author, followed by Hynynen and Kullervo (74 papers and 4,954 citations) from the University of Toronto in Canada and Yeh and Chih-Kuang (48 papers and 2,454 citations) from National Tsing Hua University in China. However, by analyzing the average citations, we find that the three authors with the highest average citation value are Hynynen, Kullervo (value = 66.95), Liu, Hao-Li (value = 60.74) and Li, Pan (value = 51.45).

**TABLE 3:** The 10 most productive authors.

Rank	Author	Institution (country)	Documents(n)	Citations	Average citations
1	Wang, Zhigang	Chongqing Medical University (China)	75	3,352	44.69
2	Hynynen, Kullervo	University of Toronto (Canada)	74	4,954	66.95
3	Yeh, Chih-Kuang	National Tsing Hua University (China)	48	2,454	51.13
4	Dayton, Paul A	University of North Carolina (USA)	46	2,111	45.89
5	Liu, Zheng	Army Medical University (China)	45	695	15.44
6	Liu, Hao-Li	National Taiwan University (China)	43	2,612	60.74
7	Kiessling, Fabian	RWTH Aachen University (Germany)	42	2027	48.26
8	Stride, Eleanor	University of Oxford (England)	42	1842	43.86
9	Li, Pan	Chongqing Medical University (China)	40	2058	51.45
10	Yan, Fei	Nanjing Medical University (China)	40	1,244	31.10

**FIGURE 4:**
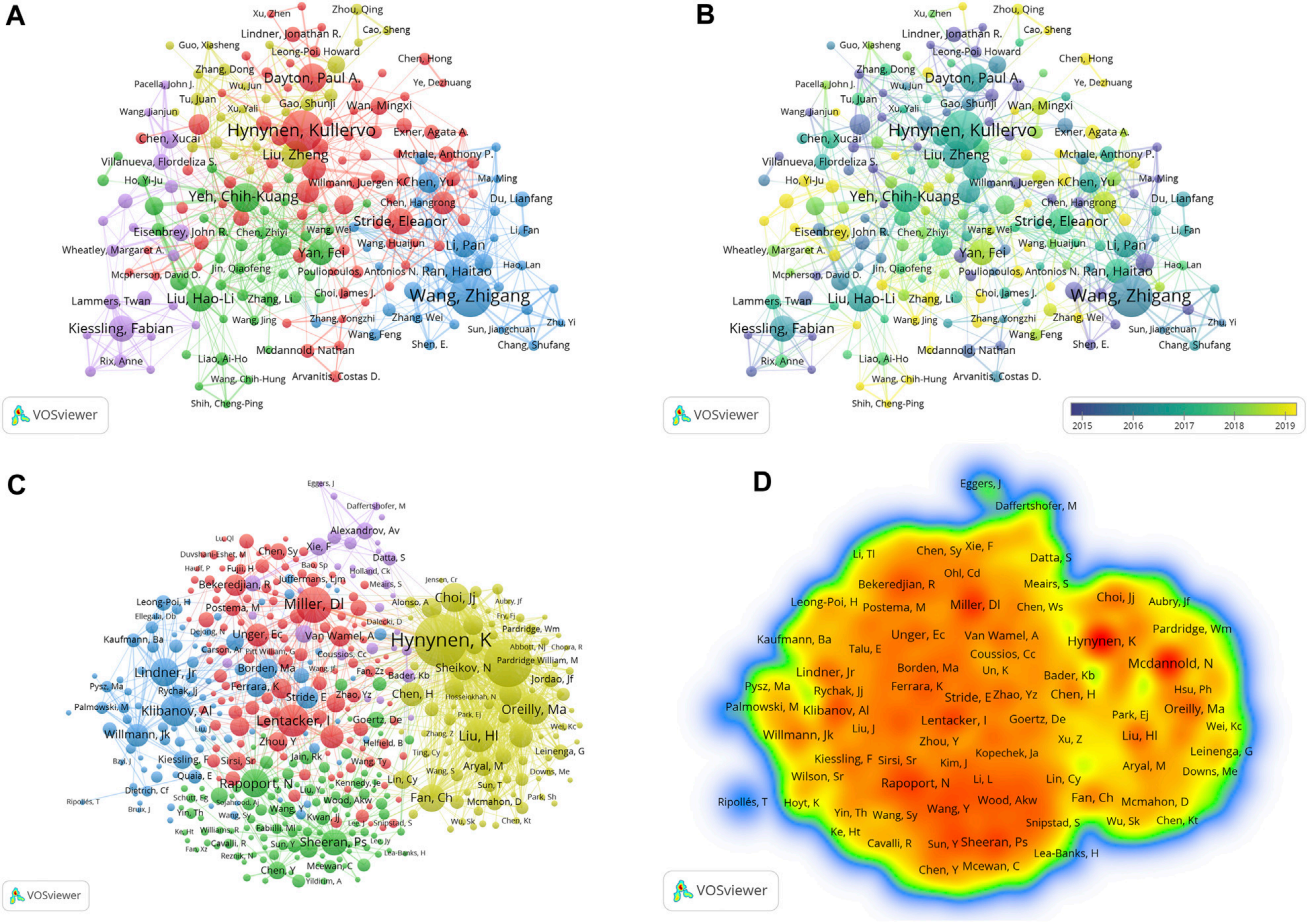
**(A)** Co-operation network between authors; **(B)** The time-overlay map of the cooperation network among the authors; **(C)** Co-operation network between co-cited authors; **(D)** VOSviewer density visualization of the co-cited authors.

The contribution of authors with a minimal productivity of 10 publications (n = 213) was visualized using VOSviewer software and shown in Figure 4A. Each node represents an author. The scale of the node represents the number of publications. The lines between the nodes represent cooperation between the authors. When combined with the superimposed visualization of the year (Figure 4B), the node colors represent the average publication years. The colder (blue) colors represent the more distant years, and the warmer (yellow) colors represent the more recent years.

Co-cited authors refer to two or more authors who are simultaneously cited through another paper. The top 10 most citations co-cited authors are listed in Table 4, and the top three are Hynynen, Kullervo (930 citations and 74,028 TLS) from the University of Toronto in Canada; McDannold, Nathan J (837 citations and 69,639 TLS) from Brigham & Women’s Hospital in the USA; and Miller, Douglas L (574 citations and 41,018 TLS) from the University of Michigan Medical School in the USA. Among them, Hynynen, Kullervo and McDannold, Nathan J have far more citations and TLS than other authors, indicating that they have made outstanding contributions and have a high academic impact in the field.

**TABLE 4:** The 10 most citations co-cited authors.

Rank	Author	Institution (country)	Citations	TLS
1	Hynynen, Kullervo	University of Toronto (Canada)	930	74,028
2	McDannold, Nathan J	Brigham & Women’s Hospital (USA)	837	69,639
3	Miller, Douglas L	University of Michigan Medical School (USA)	574	41,018
4	Lentacker, Ine	Ghent University (Belgium)	501	42,724
5	Liu, Hao-Li	National Taiwan University (China)	491	42,180
6	O'Reilly, Meaghan A	Sunnybrook Research Institute (Canada)	461	39,553
7	Klibanov, Alexander L	University of Virginia (USA)	459	39,268
8	Choi, James J	University of Oxford (England)	431	32,445
9	Lindner, Jonathan R	University of Virginia (USA)	426	31,986
10	Rapoport, Natalya	University of Utah (USA)	422	40,171

TLS: total link strength.

Figure 4C shows the co-operation network between co-cited authors with a minimal number of 50 citations (n = 452). Then, we obtained a density map of the co-cited authors by changing the unit of analysis to “density visualization” (Figure 4D). The deeper the color of a node is, the greater the number of citations.

### 3.5 Journal distribution, impact, and citation analysis

A total of 782 journals published 3,326 publications and 9,481 co-cited journals in the field of ultrasound combined with microbubble therapy technology between 2009 and 2023. Journals were ranked based on the number of articles published, with only the top 10 listed in Table 5 and the 10 most citations co-cited journals listed in Table 6. *Ultrasound in Medicine and Biology* (251 publications, 6,066 citations and 3776 TLS) was the most prolific journal, followed by the *Journal of Controlled Release* (122 publications, 7,692 citations and 3692 TLS) and *Theranostics* (96 publications, 4,311 citations and 2426 TLS). Among the top 10 co-cited journals, the top three were *Ultrasound in Medicine and Biology* (12,290 citations and 925,223 TLS), *Journal of Controlled Release* (6,870 citations and 609,344 TLS) and *Biomaterials* (3,033 citations and 262,524 TLS). In summary, compared with other journals, *Ultrasound in Medicine and Biology* and the *Journal of Controlled Release* have substantial productivity and influence in the field.

**TABLE 5:** The 10 most productive journals.

Rank	Journal	Documents(n)	Citations	TLS	Impact factor (2022)	JCR
1	Ultrasound in Medicine and Biology	251	6,066	3,776	2.9	Q2
2	Journal of Controlled Release	122	7,692	3,629	10.8	Q1
3	Theranostics	96	4,311	2,426	12.4	Q1
4	Scientific Reports	68	2041	1,176	4.6	Q2
5	Plos One	65	1825	1,089	3.7	Q2
6	Pharmaceutics	57	519	1,192	5.4	Q1
7	Ieee Transactions on Ultrasonics Ferroelectrics and Frequency Control	51	1,047	687	3.6	Q1
8	Ultrasonics Sonochemistry	49	972	613	8.4	Q1
9	Biomaterials	47	3,097	1,216	14	Q1
10	International Journal of Nanomedicine	46	1,272	727	8.0	Q1

TLS: total link strength.

**TABLE 6:** The 10 most co-cited journals.

Rank	Journal	Citations	TLS	Impact factor (2022)	JCR
1	Ultrasound in Medicine and Biology	12,290	925,223	2.9	Q2
2	Journal of Controlled Release	6,870	609,344	10.8	Q1
3	Biomaterials	3,033	262,524	14	Q1
4	Radiology	3,013	215,769	19.7	Q1
5	Journal of the Acoustical Society of America	2,842	233,300	2.4	Q2
6	Circulation	2,590	170,857	37.8	Q1
7	Theranostics	2,523	248,688	12.4	Q1
8	Ieee Transactions on Ultrasonics Ferroelectrics and Frequency Control	2,507	216,521	3.6	Q1
9	Advanced Drug Delivery Reviews	2,492	224,203	16.1	Q1
10	Proceedings of the National Academy of Sciences of the United States of America	2,188	183,965	11.1	Q1

TLS: total link strength.

**FIGURE 5:**
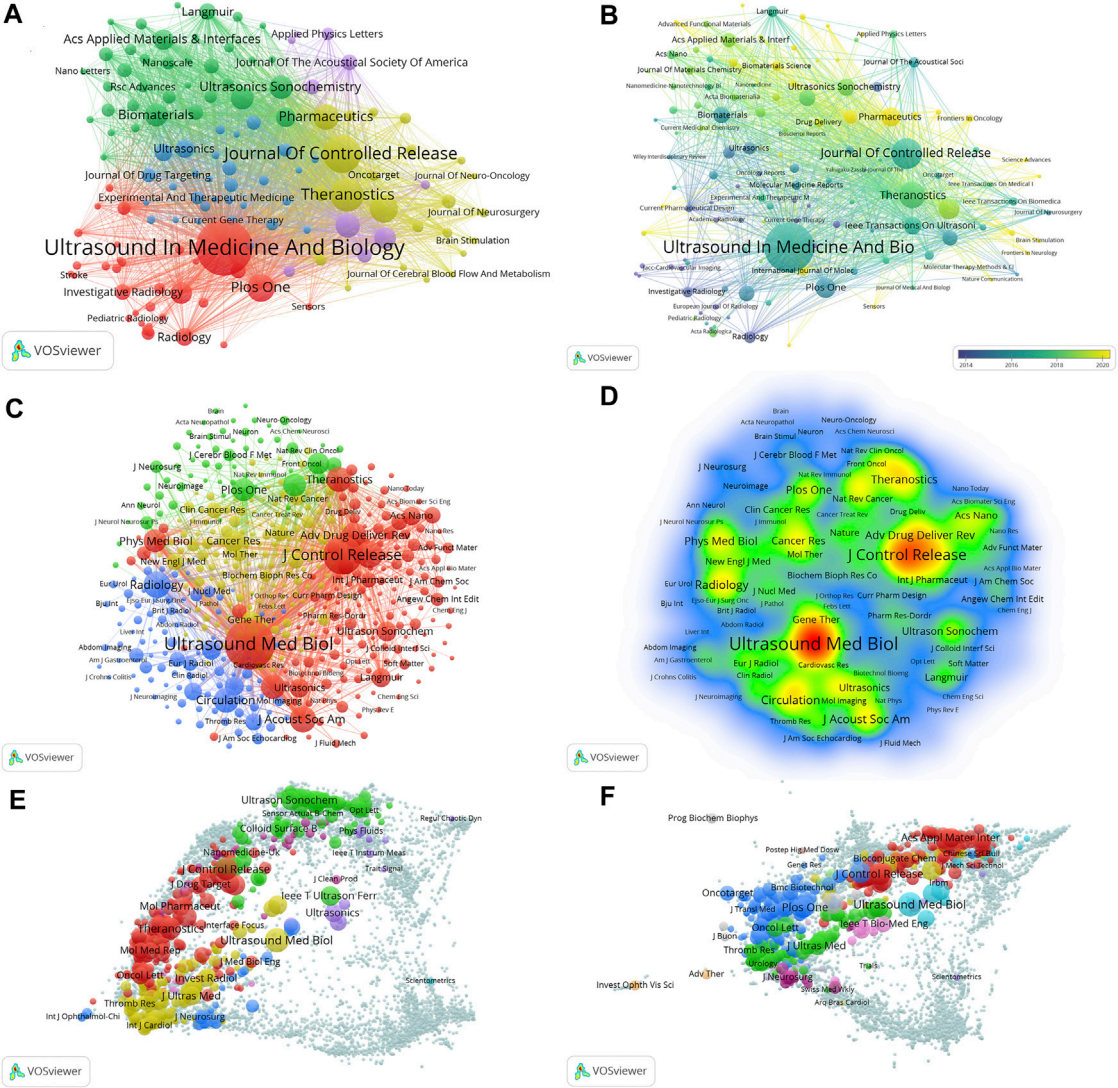
**(A)** Co-operation network between journals; **(B)** The time-overlay map of the cooperation network among the journals; **(C)** Co-operation network between co-cited journals; **(D)** VOSviewer density visualization of the co-cited journals; **(E)** The citing journals overlay map; **(F)** The co-cited journals overlay map.

The journals with a minimum productivity of 5 publications (n = 134) were visualized using VOSviewer software, and as shown in Figure 5A, their collaboration was divided into 5 clusters. By further analysis, we obtained a superimposed visualization of the year (Figure 5B). The colder (blue) colors represent the more distant years, and the warmer (yellow) colors represent the more recent years. Co-cited journals analysis was performed using VOSviewer (Figure 5C), and the density map is shown in Figure 5D. The deeper the color of a node is, the greater the number of citations. A cited map and a citing map of the journals were created by combining VOSviewer and Pajek software, as shown in Figures 5E, F.

### 3.6 Co-cited references and the strongest citation burst

We analyzed 82,835 co-cited references, and the top 10 references are listed in Table 7. The most cited article, titled “Noninvasive MR imaging-guided focal opening of the blood–brain barrier in rabbits,” was published in 2001 in the journal Radiology. Followed by titled “Ultrasound microbubble contrast agents: fundamentals and application to gene and drug delivery” was published in 2007 in the journal Annual Review of Biomedical Engineering. The third one is “Microbubbles in ultrasound-triggered drug and gene delivery,” published in Advanced Drug Delivery Reviews.

**TABLE 7:** The 10 most co-cited references.

Rank	Title	First author	Journal	Year	Citations
1	Noninvasive MR imaging-guided focal opening of the blood‒brain barrier in rabbits	Hynynen, K	Radiology	2001	324
2	Ultrasound microbubble contrast agents: fundamentals and application to gene and drug delivery	Ferrara, Katherine	Annual Review of Biomedical Engineering	2007	272
3	Microbubbles in ultrasound-triggered drug and gene delivery	Hernot, Sophie	Advanced Drug Delivery Reviews	2008	249
4	Understanding ultrasound induced sonoporation: definitions and underlying mechanisms	Lentacker, Ine	Advanced Drug Delivery Reviews	2014	191
5	Ultrasound and microbubble-targeted delivery of macromolecules is regulated by induction of endocytosis and pore formation	Meijering, Bernadet D. M	Circulation Research	2009	172
6	Temporary disruption of the blood‒brain barrier by use of ultrasound and microbubbles: safety and efficacy evaluation in rhesus macaques	Mcdannold, Nathan J	Cancer Research	2012	170
7	Vibrating microbubbles poking individual cells: drug transfer into cells via sonoporation	Van Wamel, Annemieke	Journal of Controlled Release	2006	170
8	Clinical trial of blood‒brain barrier disruption by pulsed ultrasound	Carpentier, Alexandre	Science Translational Medicine	2016	164
9	Blood‒brain barrier opening in Alzheimer’s disease using MR-guided focused ultrasound	Lipsman, Nir	Nature Communications	2018	157
10	Cellular mechanisms of the blood‒brain barrier opening induced by ultrasound in presence of microbubbles	Sheikov, Nickolai	Ultrasound in Medicine and Biology	2004	157

**FIGURE 6:**
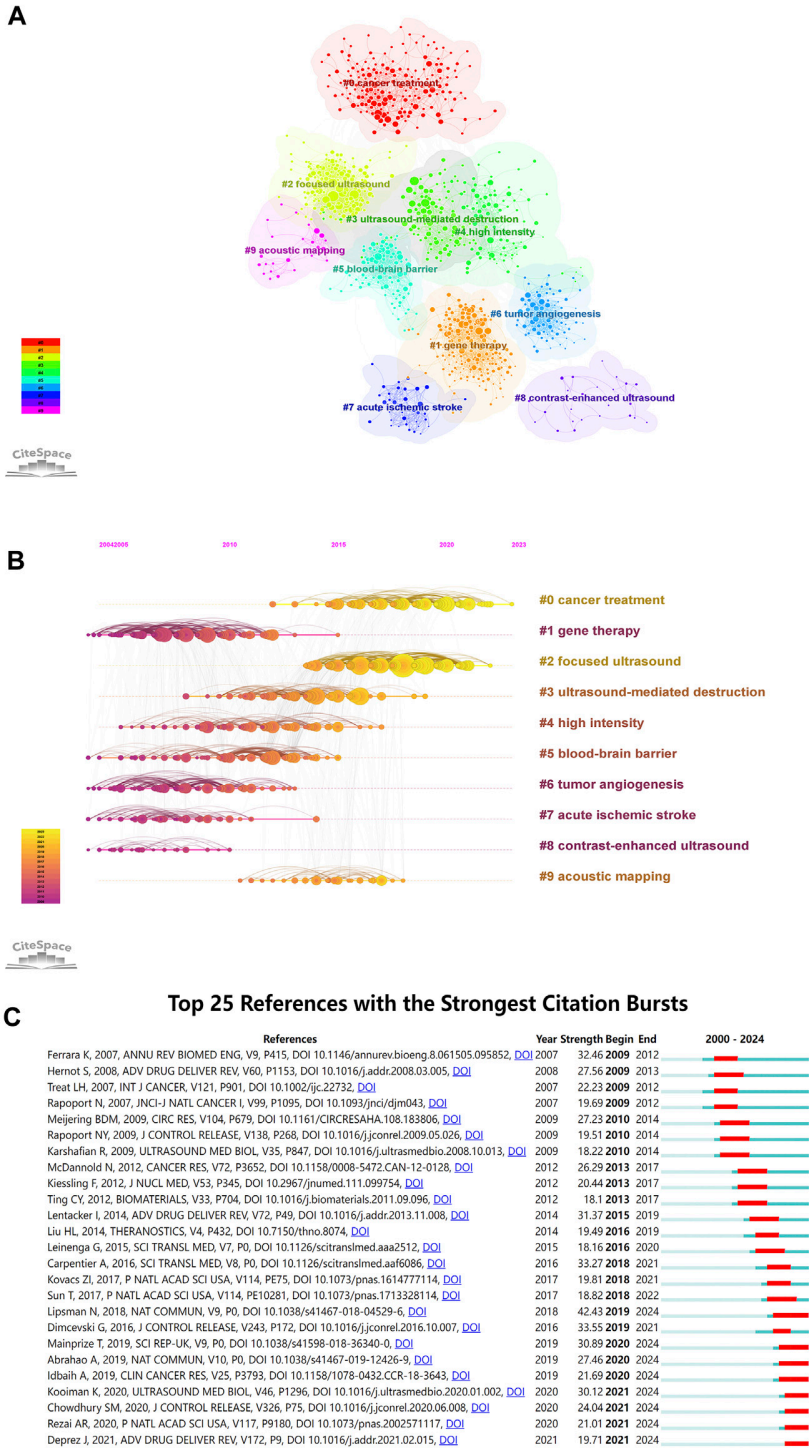
**(A)** Visualization network and cluster of co-cited references; **(B)** Timeline view map of references’ co-cited analysis; **(C)** Top 25 references with citation bursts (sorted by year the burst began).

We set associated parameters in CiteSpace to show co-citations.: “Node Types” were selected as “References,” and “Selection Criteria” was set to the top 10.0% per slice, up to 100 for references. Q values above 0.3 and S values above 0.5 suggest a stable and highly convincing clustering structure. These two indicators are key predictors of the effectiveness of graph visualization. As depicted in Figure 6A, the topic clustering map of the research field revealed 10 clusters with significant modularity and silhouette scores (Q = 0.6973; S = 0.8951), indicating a strong clustering effect and a relatively high level of homogeneity. In this study, cluster #0 cancer treatment was the largest cluster, showing a hot area for ultrasound combined with microbubble therapy technology research. Followed by #1 gene therapy, #2 focused ultrasound, and #3 ultrasound-mediated destruction. Other important clusters included #4 high intensity, #5 blood‒brain barrier, #6 tumor angiogenesis, #7 acute ischemic stroke, #8 contrast-enhanced ultrasound and #9 acoustic mapping.

A visualized timeline for these 10 clusters was further generated (Figure 6B). Each circle’s position on the horizontal axis represents the time when it first appeared, the circle’s size indicates the number of times it was cited, and the lines linking the nodes represent the co-cited relation. We found that two of them are still active, suggesting that these research directions are still under development. In addition, we also applied CiteSpace to identify the top 25 references with the strongest citation bursts (Figure 6C), which can visually present the evolution and development trend of cited references. The strength value represents the strength of citation bursts. The red bars indicate the durations of the bursts. Since 2009, the strongest citation burst has occurred in Lipsman N et al. (Lipsman et al., 2018) in 2018, followed by Dimcevski G et al. (Dimcevski et al., 2016) in 2016 and Carpentier A et al. (Carpentier et al., 2016) in 2016.

### 3.7 Keyword analysis

Keyword analysis can reveal the present research status in the field of ultrasound combined with microbubble therapy technology, including research hotspots and future development directions. In this study, there are 9,575 keywords, and we list the top 10 keywords in Table 8. The keywords “Microbubbles,” “Ultrasound” and “Therapy” ranked in the top three with 1,301, 1,092 and 738 occurrences, respectively, which was consistent with our research theme. Other important keywords included “Drug-Delivery,” “Delivery,” “Focused Ultrasound,” “Cavitation,” “Nanoparticles,” “Contrast Agents” and “Cancer.” These high-frequency keywords reflect the research trends and focus in this field.

**TABLE 8:** The 10 most occurrences keywords.

Rank	Keyword	Occurrences	TLS
1	Microbubbles	1,301	15,557
2	Ultrasound	1,092	13,434
3	Therapy	738	8,617
4	Drug-Delivery	561	6,826
5	Delivery	523	6,043
6	Focused Ultrasound	455	5,692
7	Cavitation	394	4,749
8	Nanoparticles	387	4,671
9	Contrast Agents	367	4,383
10	Cancer	363	4,340

TLS: total link strength.

**FIGURE 7:**
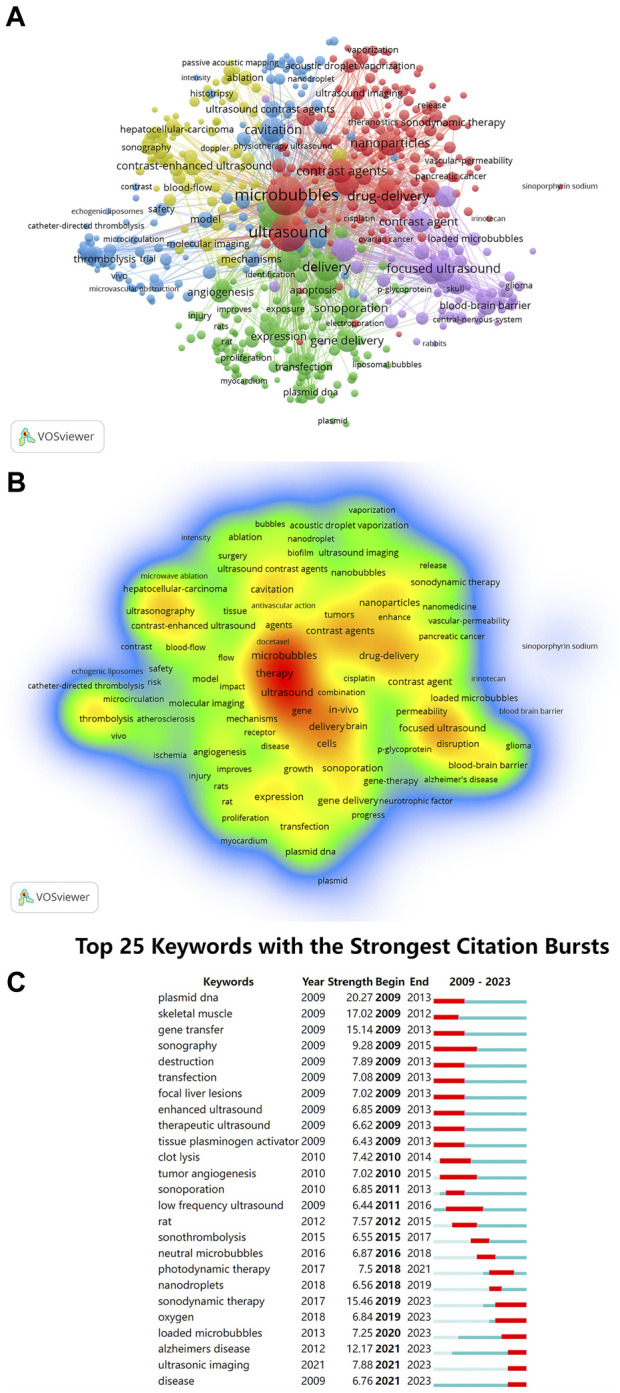
**(A)** VOSviewer cluster visualization of keywords; **(B)** VOSviewer density visualization of keywords; **(C)** Top 25 keywords with citation bursts (sorted by year the burst began).

The keywords with a minimum occurrence of 10 occurrences (n = 579) were visualized using VOSviewer software, and as shown in Figure 7A, there were 5 clusters. Furthermore, we used visualization techniques to illustrate the density of the identified keywords, as shown in Figure 7B. Keywords are mapped in color according to their relative weight, with red tones indicating higher weights. Using CiteSpace software, this study identified keywords that exhibited citation bursts and listed 25 of them, as illustrated in Figure 7C. The timeline is represented by a blue line, and the red bars show the duration of the burst, including the start and finish years, as well as the duration of the keyword burst. Notably, keywords such as “plasmid dna,” “gene transfer” and “transfection” showed that the exploration of gene treatment has always been the focus of ultrasound combined with microbubble therapy technology research in the past. However, keywords such as “sonodynamic therapy,” “loaded microbubbles” and “oxygen” have recently become popular research hotspots.

## 4 Discussion

### 4.1 General information

In this study, we used VOSviewer, CiteSpace, and the bibliometric online analysis platform to conduct a bibliometric and visual analysis of 3,326 publications related to ultrasound combined with microbubble therapy technology published between 2009 and 2023 to analyze the research status and predict future research trends in this field by examining publications, leading countries/regions, international collaborations, top institutions, major journals, authors and keywords.

The number of publications on ultrasound combined with microbubble therapy technology has been steadily increasing annually, with 2021 having the highest number of publications, thus indicating that this topic is in a steady development stage. China and the USA are the leading contributors, with more publications than other countries/regions. The USA is the most cited country, and the Netherlands has the highest average number of citations. China exhibited a high number of publications but low average citations, suggesting a demand for higher-quality publications. Furthermore, half of the top 10 institutions with the most published papers were located in China, followed by Canada, the USA and England. The University of Toronto, affiliated with Canada, had the most publications, the greatest TLS and the second highest number of average citations. Therefore, the University of Toronto has made significant contributions and impact to this field.

Among the authors, the most productive individuals were Wang, Zhigang and Kullervo Hynynen. The most citations co-cited authors were Hynynen, Kullervo and McDannold, Nathan J. We can conclude that Hynynen, Kullervo has published numerous high-quality articles and has had a significant impact in the field. Analyzing journals assists researchers in choosing suitable journals and prioritizing high-impact journals for submission while also considering their ability to reach relevant audiences and advance the field’s development. The most productive journals were *Ultrasound in Medicine and Biology*, *Journal of Controlled Release* and *Theranostics*. Moreover, the *Journal of Controlled Release* was the most cited journal, indicating that a significant number of high-quality articles were included.

### 4.2 Current research status

In modern medical applications, ultrasound has always played an important role not only as a diagnostic imaging method but also as a treatment tool (Miller et al., 2012). An increasing number of studies are exploring the application of ultrasound combined with microbubbles in the field of disease treatment (Epelbaum et al., 2022; Zhao et al., 2022; Guo et al., 2024). Since the discovery of the cavitation effect of acoustic microbubbles in the 20th century and their application in the medical field, acoustic microbubbles have been developed from the initial imaging contrast agent to the key drug of targeted therapy. According to the different cavitation effects, these materials can also be applied. Steady-state cavitation is mainly applied to enhance the effectiveness of tumor treatment and other aspects. Transient cavitation is mainly used in fields such as increasing drug uptake, opening human barriers, and disrupting tumor blood vessels. At present, there are many studies on transient cavitation, also known as ultrasound-targeted microbubble destruction technology (UTMD). UTMD is an efficient and safe technology that can effectively enhance the effectiveness of drugs in the target area (Omata et al., 2021). In conclusion, the ultrasonic-assisted microbubble therapy technique holds great potential for application.

There are various methods for using ultrasound combined with microbubble therapy for tumor treatment. Ultrasound combined with microbubbles targeted therapy technology has great potential in tumor therapy and is also a research hotspot in this field. Targeted therapy not only accurately kills tumors but also reduces the risk of tumor progression, thereby prolonging patient survival. UTMD can directly damage the microvascular wall of tumor tissue, activate the clotting mechanism and induce nutrient vascular embolism to necrotic tumors to achieve therapeutic effects (Willmann et al., 2008). This technology can be used in combination with passive targeting delivery system (PTDS), active targeting delivery system (ATDS) and biophysical targeting delivery system (BTDS) to effectively prolong the cycle time of drugs in the body and improve the efficiency of targeted therapy to improve treatment efficacy. Many scholars have conducted research on the application of the aforementioned methods (Jing et al., 2019; Liu et al., 2019; Hu et al., 2020; Li et al., 2020; Wang et al., 2022).

The BBB is a selectively permeable membrane that separates the bloodstream from the brain, aiding in safeguarding neural tissue from harmful substances (Stamp et al., 2023). However, its presence poses significant challenges for the treatment of certain diseases, as it also restricts the efficacy of drug delivery. Focused ultrasound (FUS) offers the advantages of noninvasiveness and reversible opening of the central nervous system barrier, serving as a potential noninvasive technology while also acting as a trigger for drug release (Perolina et al., 2024). FUS combined with microbubbles is one of the most researched methods for promoting BBB opening, as it interacts with nearby biological membranes, enhancing their permeability. The application of ultrasound to promote the opening of the BBB has demonstrated promising potential in a spectrum of central nervous system disorders, such as Alzheimer’s disease (Epelbaum et al., 2022; Mehta et al., 2023), amyotrophic lateral sclerosis (Abrahao et al., 2019; Shen et al., 2023), Parkinson’s disease (Fan et al., 2017; Gasca-Salas et al., 2021; Stefani et al., 2022), and central nervous system cancers (Meng et al., 2021; Sonabend et al., 2023), in current research.

Gene therapy is a biomedical approach aimed at correcting or compensating for diseases caused by genetic defects or abnormal gene expression by introducing exogenous normal genes into target cells. Ultrasound-assisted microbubble-mediated gene delivery (UMGD) is a promising nonviral gene transfer method. *In vitro* studies indicate that ultrasound-induced microbubble sonoporation can transiently increase cell membrane permeability and enhance the cellular uptake of plasmids (Sirsi and Borden, 2012; Zhou et al., 2012). *In vivo*, the application of UMGD facilitates the delivery of drugs or genetic material to various target organs for therapeutic purposes, such as the liver (Wischhusen et al., 2020; Manson et al., 2022), brain (Gorick et al., 2020), and others (Endo-Takahashi and Negishi, 2020; Wu et al., 2023). The utility of UTMD as an efficacious and secure gene transfection technique has been validated across numerous research endeavors (Wang et al., 2019; Guo et al., 2024).

In addition, ultrasound combined with microbubble therapy technology has been applied in adjunctive thrombolysis, hemostatic therapy, and sonodynamic therapy, as well as in the treatment of diseases such as diabetes, myocardial disease, renal disease and other ailments. Xie et al. utilized a combination of 2-deoxyglucose, microbubbles, and 5-aminolevulinic acid-sonodynamic therapy for the treatment of liver cancer (Xie et al., 2017). Research indicates that ultrasound-targeted microbubble destruction combined with semaglutide can effectively preserve cardiac function in rats with diabetic cardiomyopathy by substantially ameliorating myocardial fibrosis and oxidative stress (Liu et al., 2022). Ultrasound combined with microbubbles can significantly enhance local renal drug delivery, enabling low doses of methylprednisolone to ameliorate amikacin-induced renal injury in rats, thereby enhancing therapeutic efficacy (Ren et al., 2021).

### 4.3 Research hotspots and frontiers

The reference co-citation and keyword co-occurrence analyses provide valuable insights into the primary research directions and hotspots in the field of ultrasound combined with microbubble therapy from 2009 to 2023.

The most cited references are often considered foundational to research in a specific field. Co-citation analysis can help researchers identify the common knowledge bases shared by multiple studies efficiently and conveniently (Chen et al., 2014; Chen, 2017). The title “Noninvasive MR imaging-guided focal opening of the blood‒brain barrier in rabbits” was published in 2001 in the journal Radiology and is the most cited article in this field. This study concluded that the BBB can be consistently opened with focused ultrasound exposure in the presence of a US contrast agent (Hynynen et al., 2001). Notably, multiple researchers have conducted research on BBB opening/disruption at different time points and have achieved positive outcomes (Sheikov et al., 2004; McDannold et al., 2012; Carpentier et al., 2016; Lipsman et al., 2018). Ferrara, Katherine et al. published a paper titled “Ultrasound microbubble contrast agents: fundamentals and application to gene and drug delivery” in 2007 in the journal Annual Review of Biomedical Engineering (Ferrara et al., 2007). This review offers a critical analysis of the state of the art of medical microbubbles and their application in therapeutic delivery and monitoring. Several scholars have described a variety of microbubble formulations, their interaction with ultrasound and analysis of microbubble-ultrasound interactions and the potential therapeutic applications of such interactions (Hernot and Klibanov, 2008) by “Microbubbles in ultrasound-triggered drug and gene delivery,” published in 2008 in Advanced Drug Delivery Reviews.

Figure 6B shows that the current focal points of research have shifted toward #0 cancer treatment and #2 focused ultrasound. The methods for employing ultrasound combined with microbubble therapy in tumor treatment include impeding tumor vascularization and enhancing drug perfusion, among other strategies. According to existing research, both UTMD technology, which is based on the fundamental principle of transient cavitation, and the aseptic inflammatory response generated in tumor vessels under steady-state cavitation can achieve enhanced efficacy in tumor treatment. Focused ultrasound can convert ultrasound energy to specific target locations deep within the body, altering or disrupting internal tissues. Currently, focused ultrasound has been utilized in the treatment of conditions such as uterine fibroids, breast cancer, liver tumors, and Parkinson’s disease. Exploration into the application of focused ultrasound in various other ailments is also ongoing.

Based on keyword co-occurrence and cluster analysis, current research in the field of ultrasound combined with microbubble therapy primarily concentrates on ultrasound cavitation therapy (red), microbubble characteristics (yellow), drug delivery therapy (green), focused ultrasound therapy (purple), and microcirculation cavitation effects (blue). The terms “sonodynamic therapy,” “oxygen” and “loaded microbubbles” primarily refer to means or principles within the field of ultrasound combined with microbubble therapy and find application across multiple therapeutic domains, particularly in the treatment of cancer. Moreover, the keyword “Alzheimer’s disease” represents the current research focus of ultrasound combined with microbubble therapy in neurological disorders. Future research hotspots include reactive oxygen species-based sonodynamic therapy, drug-loaded microbubble targeted therapy, and Alzheimer’s disease treatment research.

Sonodynamic therapy (SDT) is a noninvasive therapeutic modality characterized by excellent tissue penetration, high precision, and minimal side effects. Targeted irradiation of lesion sites with sound-sensitive agents can elicit specific effects, such as mitochondrial dysfunction, programmed cell death, alterations in tissue or cell membrane permeability, and modulation of the immune microenvironment through physical and chemical mechanisms (Ji et al., 2021; Zhao et al., 2022). Ultrasound combined with microbubbles can significantly increase cavitation intensity and reactive oxygen species (ROS) production. In tumor treatment, ultrasound is harnessed to activate photosensitizers, generating ROS and carbon free radicals, thereby inducing tumor cell death. Compared to conventional radiotherapy and chemotherapy, it demonstrates significant superiority. A study conducted by Ji et al. presented a strategy for co-delivering O_2_ and NO to enhance SDT and reverse immune suppression, thereby enhancing the immune response against cancer (Ji et al., 2021). Furthermore, Liu first reported the antibacterial efficacy of sonodynamic therapy (SDT) against bacteria in 2011 (Liu et al., 2011). Building upon the foundation of SDT, a novel approach to antibacterial treatment has been developed. Research has shown that nanoparticle-sensitized cavitation bubbles near bacteria can cause bacterial lysis and death (Sviridov et al., 2023).

### 4.4 Limitations

This study has certain limitations. First, it exclusively incorporates literature retrieved from the WoSCC SCI-Expanded database, while articles from other databases are omitted, potentially excluding some highly influential works. Second, our focus lies in retrieving literature pertaining to ultrasound combined with microbubble therapy technology published between 2009 and 2023, thus excluding earlier publications, to discern the latest research trends and prospects in this domain. Third, despite employing various software tools to analyze the content of countries/regions, institutions, authors, keywords and references, the current methodologies are insufficient for providing a comprehensive overview of the relevant literature in this field. Moreover, the presence of multiple author identities for the same author may introduce bias into the results.

## 5 Conclusion

In conclusion, this study conducted a comprehensive bibliometric analysis and visualization of the research field concerning ultrasound combined with microbubble therapy technology. We performed a bibliometric analysis by retrieving literature relevant to this topic from the WOSCC SCIE database spanning from 2009 to 2023. China and the United States emerged as the primary contributors in this domain. In terms of publication output and global institutional collaboration, the University of Toronto in Canada has made the most significant contribution to this field. Professor Kullervo Hynynen has achieved remarkable accomplishments in this area. *Ultrasound in Medicine and Biology* is at the core of the publishing of research on ultrasound combined with microbubble therapy technology. Through analysis, we have also identified the current focal points in this field and discussed future research directions. The utilization of “reactive oxygen species-based sonodynamic therapy,” and “drug-loaded microbubble targeted therapy” for tumor treatment, along with the application of focused ultrasound to breach the BBB for treating Alzheimer’s disease, represent prominent areas of study and emerging trends in this domain. Overall, our study delved into the historical underpinnings and contemporary trends of ultrasound combined with microbubble therapy technology, providing valuable guidance for researchers.
